# Concordance between awake blood pressure on home and ambulatory blood pressure monitoring in older adults

**DOI:** 10.1093/ajh/hpag045

**Published:** 2026-05-10

**Authors:** Lama Ghazi, Emily B Levitan, Medha J Dubal, Rong Wei, Teresa N Harrison, Joseph E Schwartz, Paul Muntner, Daichi Shimbo, Kristi Reynolds

**Affiliations:** Department of Epidemiology, The University of Alabama at Birmingham, Birmingham, AL, United States; Department of Epidemiology, The University of Alabama at Birmingham, Birmingham, AL, United States; Department of Epidemiology, The University of Alabama at Birmingham, Birmingham, AL, United States; Department of Research and Evaluation, Kaiser Permanente Southern California, Pasadena, CA, United States; Department of Research and Evaluation, Kaiser Permanente Southern California, Pasadena, CA, United States; Department of Medicine, Center for Behavioral and Cardiovascular Health, Columbia University Irving Medical Center, New York, NY, United States; Department of Psychiatry and Behavioral Health, Stony Brook University Renaissance School of Medicine, Stony Brook, NY, United States; Department of Epidemiology, The University of Alabama at Birmingham, Birmingham, AL, United States; Perisphere Real World Evidence, LLC, Austin, TX, United States; Department of Medicine, Columbia University Irving Medical Center, New York, NY, United States; Department of Research and Evaluation, Kaiser Permanente Southern California, Pasadena, CA, United States; Department of Health Systems Science, Kaiser Permanente Bernard J. Tyson School of Medicine, Pasadena, CA, United States

**Keywords:** out-of-office BP measurement, ABPM, HBPM, awake BP, hypertension, blood pressure

## Abstract

**Background:**

Determining the agreement between ambulatory blood pressure (BP) monitoring (ABPM) and home BP monitoring (HBPM) can inform their use.

**Methods:**

Participants ≥65 years of age taking antihypertensive medication in the AMBROSIA-HOME study completed 24 hours of ABPM and 7 days of HBPM. Controlled office and awake BP was defined as mean systolic BP (SBP)/diastolic BP (DBP) <130/<80 mm Hg and uncontrolled office and awake BP as mean SBP or DBP ≥130/80 mm Hg. Masked uncontrolled hypertension was defined as controlled clinic BP with uncontrolled BP on HBPM or ABPM, and white coat effect as uncontrolled clinic BP with controlled BP on HBPM or ABPM.

**Results:**

Among 510 participants (mean age: 74 years; 57% female), mean (SD) awake SBP/DBP was 135.6 (13.9)/73.7 (7.9) mm Hg on HBPM and 134.7 (13.9)/73.6 (8.5) mm Hg on ABPM. The mean difference (limits of agreement) between HBPM and ABPM for awake SBP and DBP was 0.84 (−20.6 to 22.3) mm Hg and 0.07 (−12.5 to 12.6) mm Hg, respectively. Intraclass correlation coefficients for mean awake SBP and DBP between HBPM and ABPM were 0.68 (95% CI: 0.63-0.73) and 0.69 (95% CI: 0.64-0.73), respectively. The Kappa statistics (95% CI) for uncontrolled BP, masked uncontrolled hypertension, and white coat effect were 0.47 (0.39-0.55), 0.43 (0.31-0.54), and 0.35 (0.21-0.49), respectively.

**Conclusions:**

HBPM and ABPM yielded comparable mean awake BP values with a wide limit of agreement and fair to moderate agreement for identifying BP phenotypes in older adults taking antihypertensive treatment.

## Graphical abstract

**Graphical Abstract:**
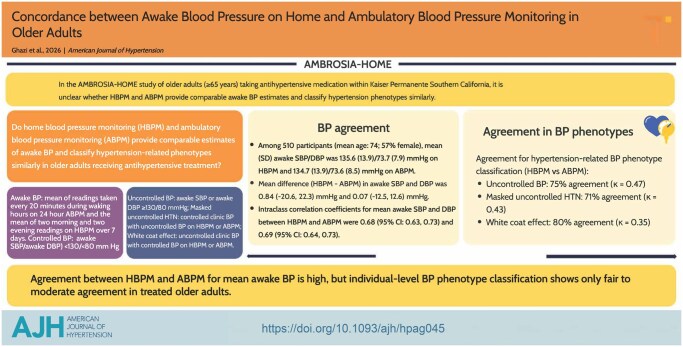

## Introduction

Out-of-office blood pressure (BP) monitoring is recommended to confirm the diagnosis of hypertension, guide antihypertensive treatment, and identify BP phenotypes associated with increased cardiovascular disease (CVD) risk.^1–3^ Ambulatory BP monitoring (ABPM) and home BP monitoring (HBPM) are the 2 modalities endorsed by the 2025 American Heart Association/American College of Cardiology (AHA/ACC) BP guideline for out-of-office BP measurement.^1^ ABPM provides a 24-hour recording with measurements taken while people are awake and asleep. A number of studies have reported higher BP on ABPM, after adjustment for office BP, to be associated with increased risk for cardiovascular outcomes.^1^^,^^4–6^ However, ABPM use remains limited in clinical practice due to its high cost, patient burden, and limited availability.^7–9^ HBPM provides measurements over a longer period, typically 7 days, with 2 measurements taken in the morning and 2 measurements in the evening while people are awake. HBPM offers a more practical and accessible approach for repeated out-of-office BP assessment, although fewer data are available on the association between BP on HBPM and cardiovascular events compared with ABPM.^1^^,^^10–13^

Although prior studies have reported inconsistent agreement between BP defined using ABPM and HBPM,^14–18^ most have focused on middle-aged adults with high office BP. Few studies have focused on older adults taking antihypertensive treatment, a growing population in whom out-of-office BP measurements may inform treatment decisions. In addition, the degree to which there is a discordance between masked uncontrolled hypertension and white coat effect when measured by ABPM and HBPM has not been well characterized in older adults.

We used data from the AMBROSIA-HOME study, a cohort of adults ≥65 years of age taking antihypertensive medication within an integrated healthcare system to assess the agreement between awake systolic BP (SBP) and diastolic BP (DBP) when measured using HBPM and ABPM. In addition, we assessed the concordance in hypertension-related BP phenotypes, including masked uncontrolled hypertension and white coat effect assessed using office BP and BP from ABPM and HBPM.

## Methods

### Study population

The AMBROSIA-HOME study design and methods have been published previously.^19^ In brief, AMBROSIA-HOME is an ancillary study to the AMBROSIA cohort, a prospective observational study of adults aged ≥65 years taking antihypertensive medication, recruited from Kaiser Permanente Southern California (KPSC) between May 2019 and November 2022. Participants attended 2 in-person study visits that were on average 1.3 days apart (range: 1-21 days). Of 670 enrolled participants, 656 completed ABPM at visit 1 and 541 initiated HBPM at visit 2. For the current analysis, we included 510 participants with complete ABPM and HBPM recordings as defined below. The protocol was approved by institutional review boards at Columbia University Irving Medical Center, University of Alabama at Birmingham, and KPSC; written informed consent was obtained from all participants.

### Study procedures

Demographic characteristics, medical history, medication use, and anthropometric data were obtained from the electronic health record (EHR). Smoking status, alcohol use, and over-the-counter medication use were collected using standardized self-reported questionnaires at visit 1.

### Research grade BP

During visit 1, research grade BP was measured on all participants. Participants were asked to sit for 5 minutes at rest, followed by 3 consecutive BP readings taken at 1-minute intervals in the dominant arm followed by 3 readings taken at 1-minute intervals in the non-dominant arm. BP was measured using an Omron Model HEM-907XL (Omron Healthcare, Inc, Lake Forest, IL), a validated oscillometric device.^20^ Mean research grade SBP and DBP was the average of all 3 readings from the non-dominant arm as this was the arm used for ABPM. Participants with mean research grade seated clinic BP ≥180/110 mm Hg or <110/50 mm Hg were referred to their healthcare clinician for evaluation and were ineligible to participate in the study.

### Routine clinic BP

All BP readings obtained as part of outpatient, non-urgent, clinical care were extracted from the EHR. We used the most recent BP prior to the study enrollment date. On average 1.6 clinic BP readings (standard deviation [SD], 1.3) were available per visit day. Attended clinic BP measurements at KPSC were performed by trained medical staff with an oscillometric device.^21^^,^^22^ When there were multiple BP readings on the same day, the average was used.^23^

### Ambulatory blood pressure monitoring

Participants were fitted with a validated SpaceLabs ABPM model 90227 device (Snoqualmie, WA) on their non-dominant arm using an appropriately sized cuff at visit 1.^24^^,^^25^ The device was programmed to record BP every 20 minutes during awake hours and every 30 minutes during sleep hours for a 24-hour period, as previously described.^19^^,^^26–28^ Participants completed a diary noting sleep and wake times, mealtimes, and any removal of the device. Awake and sleep periods were defined using these self-reports. For this analysis, we only included participants with ≥14 awake SBP and DBP readings. Mean awake SBP and DBP were calculated from all measurements during the awake period.

### Home blood pressure monitoring

HBPM was conducted using the validated Microlife WatchBP Home device (Microlife USA). Participants received the device with an appropriately sized cuff at visit 2 following the completion of ABPM. Study staff provided standardized training on proper BP measurement technique, including cuff placement, posture, and rest period. Participants demonstrated correct usage by completing 2 BP measurements during the visit and study staff confirmed the technique was correct. The HBPM device was programmed to obtain 2 consecutive BP readings, 1 minute apart, when activated. Participants were asked to measure their BP twice in the morning before breakfast and before taking antihypertensive medications and twice in the evening approximately 2 hours after dinner and before bedtime, for 7 consecutive days. For the current analysis, we included participants with complete HBPM readings defined as ≥2 morning and ≥2 evening BP readings on at least 3 of the 7 monitoring days. Mean HBPM on SBP and DBP were calculated from all morning and evening readings conducted outside of the research clinic. Participants returned the HBPM device in person or through mail.

### BP phenotype definitions

Out-of-office BP was defined using HBPM and ABPM, separately. The same BP cut-points were applied for these methods. Controlled BP was defined as mean SBP <130 mm Hg and DBP <80 mm Hg in the office and on ABPM and HBPM. Uncontrolled BP was defined as SBP ≥130 mm Hg or DBP ≥80 mm Hg.^1^ Masked uncontrolled hypertension was defined as having controlled office BP in combination with uncontrolled out-of-office BP. White coat effect was defined as having uncontrolled office BP with controlled out-of-office BP. Masked uncontrolled hypertension was assessed only among participants with controlled office BP, and white coat effect was assessed only among those with uncontrolled office BP.

### Statistical analysis

Participant characteristics were described using frequencies and percentages for categorical variables and mean and SD for continuous variables. Characteristics were reported overall and stratified by BP control status on HBPM and ABPM, separately. Histograms of SBP and DBP distributions when measured on HBPM and ABPM were generated. To evaluate agreement between HBPM and ABPM, Bland-Altman plots comparing mean awake SBP and DBP on HBPM versus ABPM were generated, with the mean difference and limits of agreement (mean difference ± 1.96 × SD) reported. Fixed bias was assessed using the mean difference, HBPM minus ABPM, and corresponding 95% confidence intervals (CI). Proportional bias was evaluated by regressing the difference, HBPM minus ABPM, on the mean of the 2 measurements. Intraclass correlation coefficients (ICCs) with 95% CIs were calculated to assess the agreement between HBPM and ABPM for mean awake SBP and DBP. We calculated the number and percentage of participants with an absolute difference in mean awake SBP and DBP between HBPM and ABPM ≥5 mm Hg and ≥10 mm Hg. We estimated the proportion of participants categorized as having uncontrolled BP, masked uncontrolled hypertension, or white coat effect based on HBPM and separately based on awake ABPM, and then compared these categorizations between the 2 methods. Agreement and reliability between out-of-office measurement methods for each BP phenotype were evaluated using overall agreement (95% CI) and Kappa statistics (95% CI). Analyses were repeated stratified by age group (65 to <73 years, ≥73 years; 73 was the median age of included participants), sex, and history of CVD, defined as an EHR-documented diagnosis of coronary heart disease or heart failure. In sensitivity analyses, agreement and reliability of uncontrolled BP, masked uncontrolled hypertension, or white coat effect were estimated using HBPM and ABPM with office BP defined by measurements extracted from the EHR, rather than research study BP.

In sensitivity analyses, agreement and reliability of uncontrolled BP, masked uncontrolled hypertension, and white coat effect were re-evaluated using an alternative BP threshold of 140/90 mm Hg, stage 2 hypertension in the 2025 AHA/ACC BP guideline, for research-grade BP and 135/85 mm Hg for HBPM and awake ABPM.

All analyses were performed using SAS version 9.4 (SAS Institute, Cary, NC, USA).

## Results

### Participant characteristics

Among 510 participants, mean (SD) age was 74.3 (6.1) years, 56.9% were female, and 18.0% and 21.8% were Hispanic and non-Hispanic Black, respectively (Table 1). A majority of participants reported alcohol use in the past year (61.6%). Arthritis (46.3%) and diabetes (29.6%) were the most common comorbidities. The median [25th, 75th percentiles] number of antihypertensive medication classes being taken was 2 [1, 2]. Mean (SD) research grade SBP/DBP was 132.1 (15.4)/69.8 (10.1) mm Hg, while awake SBP/DBP was 135.6 (13.9)/73.7 (7.9) mm Hg by HBPM and 134.7 (13.9)/73.6 (8.5) mm Hg by ABPM (Table 2). Compared to participants with white coat effect, those with masked uncontrolled hypertension defined using HBPM (Table S1) and ABPM (Table S2) were older and less likely to be non-Hispanic Black participants.

**Table 1:** Characteristics of AMBROSIA-HOME participants overall and among those with controlled and uncontrolled BP on ABPM and HBPM.

		HBPM		ABPM	
	Total, *n = *510	Controlled BP, *n = *180	Uncontrolled BP, *n = *330	Controlled BP, *n = *185	Uncontrolled BP, *n = *325
Demographics and anthropometrics					
Age in years, mean (SD)	74.3 (6.1)	73.5 (5.7)	74.7 (6.3)	73.9 (5.9)	74.5 (6.2)
Female, *n* (%)	290 (56.9%)	109 (60.6%)	181 (54.9%)	100 (54.1%)	190 (58.5%)
Race/ethnicity, *n* (%)					
Non-Hispanic White	230 (45.1%)	91 (50.6%)	139 (42.1%)	88 (47.6%)	142 (47.6%)
Non-Hispanic Black	111 (21.8%)	34 (18.9%)	77 (23.3%)	41 (22.2%)	70 (21.5%)
Hispanic	92 (18.0%)	31 (18.9%)	61 (18.5%)	32 (17.3%)	60 (18.5%)
Asian/Pacific Islander	68 (13.3%)	22 (12.2%)	46 (13.9%)	23 (12.4%)	45 (13.9%)
Other/unknown	9 (1.8%)	2 (1.1%)	7 (2.12%)	1 (0.5%)	8 (2.5%)
Body mass index in kg/m^2^, mean (SD)	28.9 (5.3)	28.8 (5.1)	28.9 (5.4)	29.2 (5.3)	28.8 (5.3)
Social history					
Ever smoker, *n* (%)	196 (38.4%)	63 (35.0%)	133 (40.3%)	76 (41.1%)	120 (36.9%)
Drank alcohol in the past year, *n* (%)	314 (61.6%)	109 (60.6%)	205 (62.1%)	105 (56.8%)	209 (64.3%)
Medical history					
Diabetes mellitus, *n* (%)	151 (29.6%)	41 (22.8%)	110 (33.3%)	48 (25.9%)	103 (31.7%)
Arthritis, *n* (%)	236 (46.3%)	70 (38.9%)	166 (50.3%)	73 (39.5%)	163 (50.2%)
Parkinson disease, *n* (%)	4 (0.8%)	0 (0.0%)	4 (1.2%)	0 (0.0%)	4 (1.2)
Neuropathy, *n* (%)	52 (10.2%)	14 (7.8%)	38 (11.5%)	19 (10.3%)	33 (10.2%)
Coronary heart disease, *n* (%)	87 (17.1%)	26 (14.4%)	61 (18.5%)	33 (17.8%)	54 (16.6%)
Heart failure, *n* (%)	20 (3.9%)	7 (3.9%)	13 (3.9%)	8 (4.3%)	12 (3.7%)
Depression, *n* (%)	98 (19.2%)	34 (18.9%)	64 (19.4%)	41 (22.2%)	57 (17.5%)
Chronic kidney disease, *n* (%)	117 (22.9%)	39 (21.7%)	78 (23.6%)	44 (23.8%)	73 (22.5%)
Medications					
Antihypertensive medication classes, median [25, 75th percentiles]	2 [1, 2]	2 [1, 3]	2 [1, 2]	2 [1, 3]	2 [1, 2]

Controlled BP defined as SBP <130 mm Hg and DBP <80 mm Hg; uncontrolled BP defined as SBP ≥130 mm Hg or DBP ≥80 mm Hg; chronic kidney disease: estimated glomerular filtration rate <60 mL/min/1.73 m^2^.

Abbreviations: ABPM, ambulatory blood pressure monitoring; BP, blood pressure; HBPM, home blood pressure monitoring; IQR, interquartile range; SD, standard deviation.

**Table 2:** Blood pressure of participants overall and among those with controlled and uncontrolled hypertension on ABPM and HBPM.

	Overall, *n = *510	Controlled BP on HBPM, *n = *180	Uncontrolled BP on HBPM, *n = *330	Controlled BP on ABPM, *n = *185	Uncontrolled BP on ABPM, *n = *325
Systolic blood pressure in mm Hg, mean (SD)					
Research grade	131.1 (14.9)	123.0 (12.1)	135.5 (14.4)	123.5 (12.2)	135.5 (14.5)
Routine clinic	133.0 (12.6)	127.9 (11.6)	135.8 (12.2)	129.0 (12.4)	135.3 (12.2)
Awake HBPM	135.6 (13.9)	121.9 (6.4)	142.9 (10.9)	126.2 (9.6)	140.9 (13.1)
Awake ABPM	134.7 (13.9)	124.8 (10.3)	140.2 (12.5)	121.2 (6.7)	142.4 (10.6)
Diastolic blood pressure in mm Hg, mean (SD)					
Research grade	68.8 (10.2)	67.2 (10.0)	69.7 (10.3)	66.5 (9.5)	70.2 (10.4)
Routine clinic	70.2 (9.7)	68.6 (9.6)	71.1 (9.7)	68.6 (9.5)	71.1 (9.7)
Awake HBPM	73.7 (7.9)	68.9 (5.7)	76.2 (7.8)	70.5 (5.9)	75.4 (8.4)
Awake ABPM	73.6 (8.5)	70.9 (7.5)	75.0 (8.6)	68.6 (6.3)	76.4 (8.3)

Controlled BP defined as SBP <130 mm Hg and DBP <80 mm Hg; uncontrolled BP on HBPM defined as SBP ≥130 mm Hg or DBP ≥80 mm Hg.

Abbreviations: ABPM, ambulatory blood pressure monitoring; BP, blood pressure; EHR: electronic health record; HBPM, home blood pressure monitoring; SD, standard deviation.

### Agreement between HBPM and ABPM

**Figure 1:**
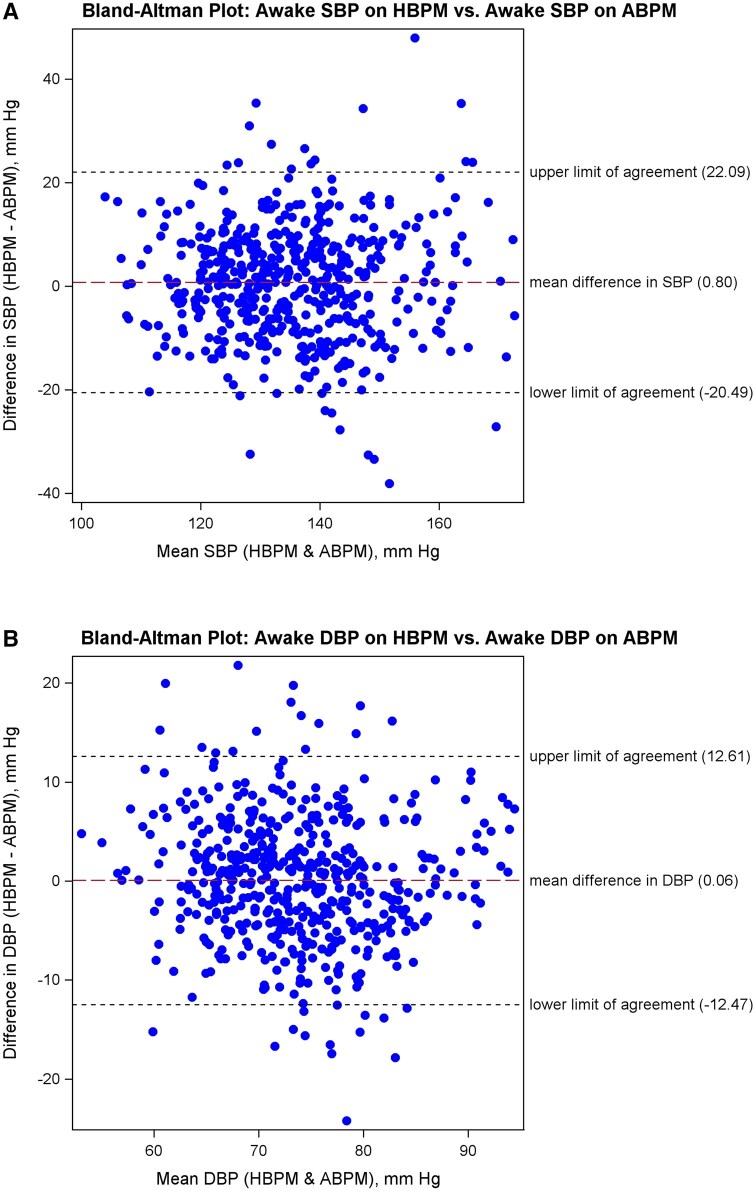
(A) Bland-Altman plot comparing awake SBP from HBPM to awake SBP from ABPM. Solid line represents the mean difference in awake SBP (0.84 mm Hg, 95% CI: −0.11 to 1.79) and dashed lines show the limits of agreement (±1.96 × standard deviation of difference). Bland-Altman plot indicates that for approximately 95% of participants, the difference between the 2 measures of awake SBP (HBPM-ABPM) is between −20.65 and 22.33 mm Hg. (B) Bland-Altman plot comparing awake DBP from HBPM to awake SBP from ABPM. Solid line represents the mean difference in awake DBP (0.07 mm Hg, 95% CI: −0.48 to 0.63) and dashed lines show the limits of agreement (±1.96 × standard deviation of difference). Bland-Altman plot indicates that for approximately 95% of participants, the difference between the 2 measures of awake DBP (HBPM-ABPM) is between −12.48 to 12.63 mm Hg. Abbreviations: ABPM, ambulatory blood pressure monitoring; DBP, diastolic blood pressure; HBPM, home blood pressure monitoring; SBP, systolic blood pressure.

**Figure 2:**
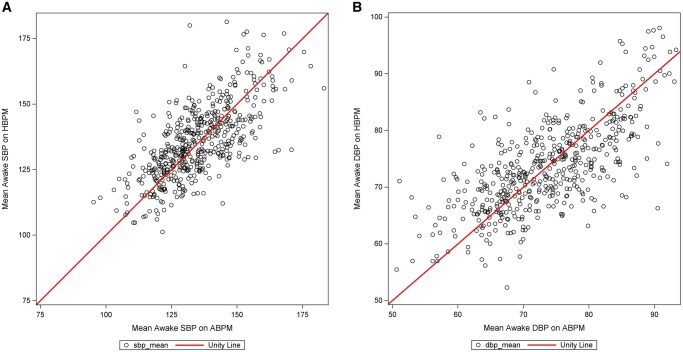
(A) Scatter plot of mean awake SBP on ABPM vs. HBPM. The red line represents the unity line (perfect agreement). Intraclass correlation coefficient (ICC) and 95% CI: 0.68 (0.63-0.73). (B) Scatter plot of mean awake DBP on ABPM vs. HBPM. The red line represents the unity line (perfect agreement). Intraclass correlation coefficient (ICC) and 95% CI: 0.69 (0.64-0.73). Abbreviations: ABPM, ambulatory blood pressure monitoring; DBP, diastolic blood pressure; HBPM, home blood pressure monitoring; SBP, systolic blood pressure.

The mean difference in awake BP between HBPM and ABPM was 0.84 mm Hg (95% CI: −0.11 to 1.79) for SBP and 0.07 mm Hg (95% CI: −0.48 to 0.63) for DBP. Bland-Altman plots (Figure 1A and B) show that the limits of agreement for the difference in awake SBP between HBPM and ABPM were −20.7 to 22.3 mm Hg, and for awake DBP were −12.5 to 12.6 mm Hg. The distribution of differences between HBPM and ABPM measurements are shown in Figure S1A for SBP and Figure S1B for DBP. In regression analyses assessing proportional bias, the slope for SBP was 0.002 (95% CI: −0.073 to 0.076, *P* = .967) and for DBP was −0.083 (95% CI: −0.156 to −0.008, *P* = .028). The ICC between HBPM and ABPM was 0.68 (95% CI: 0.63 to 0.73) for SBP and 0.69 (95% CI: 0.64 to 0.73) for DBP (Figure 2A and B, respectively). Overall, 34.9% of participants had an absolute difference in mean SBP ≥5 mm Hg when comparing HBPM to ABPM, and 17.1% had an absolute difference in mean SBP ≥10 mm Hg. When comparing mean awake DBP on HBPM to ABPM, 21.9% of participants had a mean absolute difference ≥5 mm Hg and 5.1% had a mean absolute difference ≥10 mm Hg (Table S3).

The prevalence of uncontrolled BP was 64.7% on HBPM and 66.5% on ABPM. Overall agreement between HBPM and ABPM for uncontrolled BP was 75% (95% CI: 72-79), with a kappa of 0.47 (95% CI: 0.39-0.55) (Table 3). Among those with controlled office BP (*n = *230), 48% (*n = *111) and 47% (*n = *109) had masked uncontrolled hypertension by HBPM and ABPM, respectively. Overall agreement between HBPM and ABPM for masked uncontrolled hypertension was 71% (95% CI: 66-77), with a kappa of 0.43 (95% CI: 0.31-0.54). Among those with uncontrolled research grade BP (*n = *280), 22% (*n = *61) had white coat effect when defined on HBPM and 23% (*n = *64) on ABPM. Overall agreement for white coat effect was 80% (95% CI: 75-84), with a kappa of 0.35 (95% CI: 0.21-0.49). When stratified by age group, agreement estimates showed point estimates falling within each other’s 95% CIs across the 2 age subgroups (65 to <73 years and ≥73 years), sex, and history of CVD (Table S4).

**Table 3:** Agreement of HBPM and ABPM in identifying uncontrolled blood pressure, masked uncontrolled hypertension and white coat effect.

Uncontrolled hypertension			
	ABPM—yes	ABPM—no	Total
HBPM—yes	266	64	330
HBPM—no	59	121	180
Total	325	185	510
Overall agreement (95% CI):			0.75 (0.72-0.79)
Kappa (95% CI):			0.47 (0.39-0.55)

Masked uncontrolled hypertension			
	ABPM—yes	ABPM—no	Total
HBPM—yes	82	36	118
HBPM—no	35	97	132
	117	133	250
Overall agreement (95% CI):			0.71 (0.66-0.77)
Kappa (95% CI):			0.43 (0.31-0.54)

White coat effect			
	ABPM—yes	ABPM—no	Total
HBPM—yes	24	24	48
HBPM—no	28	184	212
Total	52	208	260
Overall agreement (95% CI):			0.8 (0.75-0.84)
Kappa (95% CI):			0.35 (0.21-0.49)

Uncontrolled BP defined as SBP ≥130 mm Hg or DBP ≥80 mm Hg; masked uncontrolled BP defined as research grade SBP <130 mm Hg and research grade DBP <80 mm Hg and mean out-of-office SBP ≥130 mm Hg or mean out-of-office DBP ≥80 mm Hg; white coat effect defined as research grade SBP ≥130 mm Hg or research grade DBP ≥80 mm Hg and mean out-of-office SBP <130 mm Hg and mean out-of-office DBP <80 mm Hg.

Abbreviations: ABPM, ambulatory blood pressure monitoring; BP, blood pressure; CI: confidence interval; HBPM, home blood pressure monitoring.

### Sensitivity analyses

Among participants with controlled routine clinic BP (*n = *171), 49% had masked uncontrolled hypertension by HBPM and 50% by ABPM. Overall agreement between methods for masked uncontrolled hypertension was 70% (95% CI: 63-77) with a kappa statistic of 0.40 (95% CI: 0.26-0.54). Among participants with uncontrolled routine clinic BP (*n = *339), the prevalence of white coat effect was 27% by HBPM and 29% by ABPM. Overall agreement for white coat effect was 78% (95% CI: 74-83) with a kappa of 0.47 (95% CI: 0.37-0.58) (Table S5). Using routine clinic BP, agreement estimates showed point estimates falling within each other’s 95% CIs within each age, sex, and CVD history sub-group (Table S6). When applying a cut-point of 140/90 mm Hg for research BP and 135/85 mm Hg for HBPM and awake ABPM, compared to the primary analysis the prevalence of uncontrolled BP was lower (1.4% on HBPM and 1.0% on ABPM) and the prevalence of masked uncontrolled hypertension and white coat effect also declined. Kappa statistics were 0.83 (95% CI: 0.60-1.00) for uncontrolled BP, 0.38 (95% CI: 0.26-0.51) for masked uncontrolled hypertension, and 0.46 (95% CI: 0.30-0.62) for white coat effect (Table S7).

## Discussion

There are several key findings from this study evaluating the agreement between HBPM and ABPM for assessing awake BP phenotypes in older adults. While mean awake SBP and DBP were comparable on HBPM and ABPM, large individual-level differences were present, and the prevalence of BP phenotypes was similar by both methods yet individual-level agreement was fair to moderate and many participants were categorized differently by the 2 methods.

Prior studies comparing HBPM and ABPM have shown modest differences in mean SBP, but often poor agreement in phenotype categorization, with results varying across populations and clinical settings. In the Swiss Longitudinal Cohort Study of middle-aged and older adults (mean age: 61 years) with suspected or treated hypertension, daytime SBP was 6.9 mm Hg higher on ABPM than HBPM, and white-coat hypertension was more common using ABPM compared to HBPM (28% vs. 4.3%).^14^ Similarly, in the Japan Morning Surge Home Blood Pressure (J-HOP) study which enrolled Japanese adults (mean age: 65 years) taking antihypertensive medication, mean SBP was 8.8 mm Hg higher when assessed by ABPM compared with HBPM.^15^ Additionally, in a European cohort (mean age: 57 years), only 48% of individuals with masked hypertension had concordant elevations on both HBPM and ABPM, with the remainder having isolated ambulatory (30%) or isolated home (22%) masked hypertension.^29^ The individual-level discordance likely reflects inherent differences in how each method captures BP: ABPM measures BP episodically throughout waking hours including during physical activity and periods when stressful events may be occurring, whereas HBPM guidelines recommend measurements to be conducted while in a seated position. These contextual differences, along with measurement frequency and differences in device algorithms, likely contribute to the discordance in phenotype classification. Future studies that include more frequent home BP measurements during awake and sleep periods or that compare phenotype classification using intent-to-treat approaches, may clarify the extent to which measurement frequency and duration affect agreement between the 2 methods.

The clinical relevance of agreement between HBPM and ABPM lies in their use for risk stratification and treatment decisions. Although the present study did not evaluate outcomes, prior research has shown that HBPM has a stronger association with target organ damage and cardiovascular events than office BP, with similarly strong association as ABPM. Therefore, both out-of-office methods may offer important clinical value.^1^^,^^30–36^ These data support HBPM as a meaningful measure of out-of-office BP, complementary to ABPM, particularly when long-term monitoring or repeated assessments are needed. HBPM and ABPM may capture different BP constructs, with ABPM reflecting BP over 24 hours, including activity and sleep, and HBPM BP while resting. In this context, the current findings show that ABPM and HBPM capture different phenotypes. Thus, the choice between HBPM and ABPM should be guided by clinical context. ABPM remains the standard for obtaining sleep BP and circadian patterns. HBPM, while less comprehensive, is more widely available, lower in cost, better tolerated, and enables repeated long-term monitoring. For patients in whom masked uncontrolled hypertension or white coat effect is suspected and ABPM is not available or feasible, HBPM is a practical alternative, though clinicians should be aware of the individual-level discordance documented in the current and prior studies. The 2025 AHA/ACC guideline endorses both modalities for out-of-office BP assessment and recommends confirmatory testing when clinical and out-of-office BP values are discordant.^1^ Our findings reinforce this recommendation, particularly for older adults where precise phenotyping carries significant implications for treatment decisions. Whether BP phenotype discordance between HBPM and ABPM affects treatment decisions and cardiovascular outcomes warrants evaluation in future longitudinal research.

This study has several strengths. The AMBROSIA-HOME cohort included a diverse sample of older adults recruited from an integrated health system with standardized hypertension management practices, enhancing generalizability to real-world populations aged ≥65 years taking antihypertensive medication.^23^^,^^37^ Both ABPM and HBPM were implemented using validated devices and standardized protocols, and BP phenotypes were defined using both research-grade and routine clinic BP values. However, certain limitations should be considered. First, although participants were trained in how to use the HBPM device and take a proper reading, adherence to the instructions could not be verified. Second, different devices were used for ABPM and HBPM. Although both devices have been validated, their measurement algorithms and calibration may have contributed to observed discrepancies. Third, the monitoring order was not randomized; all participants completed ABPM before HBPM, potentially introducing carryover effects. Additionally, more participants had missing HBPM data (*n = *131) than ABPM data (*n = *14), as HBPM was added after initial enrollment began; we cannot exclude the possibility that this differential exclusion introduced selection bias. Fourth, research grade BP and routine-clinic BP were obtained from a single visit rather than the multi-visit assessment for phenotype classification. Fifth, the modest number of participants with white coat effect limits precision for agreement estimates in this subgroup. Sixth, we did not have data to compare the prognostic clinical utility of mean SBP and DBP or BP phenotypes defined by ABPM versus HBPM. Finally, given the study’s focus on older adults enrolled in a comprehensive healthcare system, findings may not be fully generalizable to younger individuals, those untreated for hypertension, or those in less-integrated care settings.

In this study of older adults taking antihypertensive medication, HBPM and ABPM yielded similar mean awake BP values, with mean differences of less than 1 mm Hg for both SBP and DBP and intraclass correlation coefficients of 0.68 and 0.69 for SBP and DBP, respectively, indicating moderate agreement on a continuous scale. However, individual-level agreement for identifying hypertension-related BP phenotypes was fair to moderate (kappa: 0.35-0.47), with many participants categorized differently by the 2 methods. These findings suggest that while HBPM and ABPM produce comparable average awake BP values for a population, meaningful differences exist for individuals that may impact treatment recommendations. These results should be interpreted in the context of the study’s limitations, including the non-randomized order of device use and the inability to verify HBPM adherence to standardized protocols.

## Supplementary Material

hpag045_Supplementary_Data

## Data Availability

The data underlying this article can be shared on reasonable request to the corresponding author and approval by AMBROSIA-HOME study group.
