# Supplementary material for: Concordance between awake blood pressure on home and ambulatory blood pressure monitoring in older adults
Source: Am J Hypertens. 2026 May 10;39(9):1083–91. doi: 10.1093/ajh/hpag045 (PMC13473872; doi:10.1093/ajh/hpag045)
Supplement: Supplementary file 1 [file hpag045_supplementary_data.docx]

**SUPPLEMENTAL MATERIALS

Concordance between Awake Blood Pressure on Home and Ambulatory Blood Pressure Monitoring in Older Adults**

**Authors**: Lama Ghazi^a^, Emily B. Levitan^a^, Medha J. Dubal^a^, Rong Wei^b^, Teresa N. Harrison^b^, Joseph E. Schwartz^c,d^, Paul Muntner ^a,e^, Daichi Shimbo^f^, Kristi Reynolds^g,h^
 ^a^ Department of Epidemiology, The University of Alabama at Birmingham, Birmingham, AL

^b^ Department of Research & Evaluation, Kaiser Permanente Southern California, Pasadena, CA

^c^ Department of Medicine, Center for Behavioral and Cardiovascular Health, Columbia University Irving Medical Center, New York, NY

^d^ Department of Psychiatry and Behavioral Health, Stony Brook University Renaissance School of Medicine, Stony Brook, NY

^e^ Perisphere Real World Evidence, LLC, Austin, TX

^f^ Department of Medicine, Columbia University Irving Medical Center, New York, NY

^g^ Department of Research & Evaluation, Kaiser Permanente Southern California, Pasadena, CA
^h^ Department of Health Systems Science, Kaiser Permanente Bernard J Tyson School of Medicine, Pasadena, CA

**Short title**: HBPM versus ABPM in older adults

**Corresponding author**:
Lama Ghazi, MD PhD
Department of Epidemiology, School of Public Health
University of Alabama at Birmingham
1665 University Blvd, Birmingham, AL 35233
Phone: 205-975-5792 | Email: [lghazi@uab.edu](mailto:lghazi@uab.edu)

**Table S1. Characteristics of AMBROSIA-HOME Participants by BP phenotypes as defined by HBPM**

|  | **Masked uncontrolled hypertension  n= 111** | **White coat effect n= 61** |
| --- | --- | --- |
| **Demographics and anthropometrics** | | |
| Age in years, mean (SD) | 75.7 (6.9) | 73.0 (4.8) |
| Female, n (%) | 55 (49.6) | 41 (67.2) |
| Race/Ethnicity, n (%) | | |
| Non-Hispanic White | 60 (54.1) | 28 (45.9) |
| Non-Hispanic Black | 15 (13.5) | 15 (24.6) |
| Hispanic | 19 (13.5) | 10 (16.4) |
| Asian/Pacific Islander | 16 (14.4) | 7 (11.5) |
| Other/Unknown | 1 (0.9) | 1 (1.6) |
| Body mass index in kg/m^2^, mean (SD) | 29.0 (5.3) | 28.5 (5.0) |
| **Social history** | | |
| Ever smoker, n (%) | 49 (48.1) | 16 (26.2) |
| Drank alcohol in the past year, n (%) | 66 (59.8) | 39 (63.9) |
| **Medical history** | | |
| Diabetes mellitus, n (%) | 27 (24.3) | 17 (27.9) |
| Arthritis, n (%) | 52 (46.9) | 23 (37.7) |
| Parkinson disease, n (%) | 1 (0.9) | 0 (0.0) |
| Neuoropathy, n (%) | 11 (9.9) | 6 (9.8) |
| Coronary heart disease, n (%) | 26 (23.4) | 8 (13.1) |
| Heart failure, n (%) | 3 (2.7) | 2 (3.3) |
| Depression, n (%) | 23 (20.7) | 8 (13.1) |
| Chronic kidney disease, n (%) | 27 (24.3) | 13 (21.3) |
| **Medications** | | |
| Antihypertensive medication classes, median [IQR] | 2 [1-3] | 2 [2-3] |
| **Systolic Blood Pressure in mm Hg, mean (SD)** | | |
| Research grade | 120.5 (5.9) | 137.2 (8.7) |
| Routine clinic | 132.3 (11.7) | 131.6 (11.5) |
| Awake HBPM | 139.8 (8.7) | 123.1 (7.7) |
| Awake ABPM | 135.5 (10.0) | 127.2 (11.7) |
| Asleep ABPM | 122.2 (13.1) | 111.9 (11.9) |
| 24-hour ABPM | 130.9 (9.4) | 121.6 (10.1) |
| **Diastolic Blood Pressure in mm Hg, mean (SD)** | | |
| Research grade | 64.2 (7.4) | 74.2 (9.0) |
| Routine clinic | 71.1 (9.7) | 71.1 (9.4) |
| Awake HBPM | 75.2 (7.0) | 69.1 (6.3) |
| Awake ABPM | 72.8 (8.4) | 71.9 (8.8) |
| Asleep ABPM | 63.4 (7.9) | 62.4 (8.1) |
| 24-hour ABPM | 69.6 (7.3) | 68.4 (7.8) |
| BP: blood pressure; HBPM: home blood pressure monitoring; SD: standard deviation; IQR: interquartile range; Chronic kidney disease: estimated Glomerular Filtration rate <60 mL/min/1.73m2 | | |

**Table S2. Characteristics of AMBROSIA-HOME Participants by BP phenotypes as defined by ABPM**

|  | **Masked uncontrolled hypertension  n= 109** | **White coat effect n= 64** |
| --- | --- | --- |
| **Demographics and anthropometrics** | | |
| Age in years, mean (SD) | 75.2 (6.8) | 73.4 (5.2) |
| Female, n (%) | 57 (52.3) | 34 (53.1) |
| Race/Ethnicity, n (%) | | |
| Non-Hispanic White | 55 (50.5) | 20 (31.3) |
| Non-Hispanic Black | 15 (13.8) | 22 (34.4) |
| Hispanic | 22 (20.2) | 14 (21.9) |
| Asian/Pacific Islander | 16 (14.7) | 8 (12.5) |
| Other/Unknown | 1 (0.9) | 0 (0.0) |
| Body mass index in kg/m^2^, mean (SD) | 29.0 (5.1) | 29.63 (5.2) |
| **Social history** | | |
| Ever smoker, n (%) | 42 (38.5) | 22 (34.4) |
| Drank alcohol in the past year, n (%) | 68 (62.4) | 37 (57.8) |
| **Medical history** | | |
| Diabetes mellitus, n (%) | 25 (22.9) | 22 (34.4) |
| Arthritis, n (%) | 51 (46.8) | 25 (39.1) |
| Parkinson disease, n (%) | 1 (0.9) | 0 (0.0) |
| Neuoropathy, n (%) | 6 (5.5) | 6 (9.4) |
| Coronary heart disease, n (%) | 22 (20.2) | 11 (17.2) |
| Heart failure, n (%) | 2 (1.8) | 2 (3.1) |
| Depression, n (%) | 19 (17.4) | 11 (17.2) |
| Chronic kidney disease, n (%) | 23 (21.1) | 14 (21.9) |
| **Medications** | | |
| Antihypertensive medication classes, median [IQR] | 2 (1) | 2 (1.5) |
| **Systolic Blood Pressure in mm Hg, mean (SD)** | | |
| Research grade | 120.6 (5.9) | 138.8 (10.01) |
| Routine clinic | 132.1 (11.1) | 134.3 (11.60) |
| Awake HBPM | 136.4 (11.8) | 129.0 (10.43) |
| Awake ABPM | 138.3 (8.2) | 121.4 (7.18) |
| Asleep ABPM | 123.7 (13.1) | 110.6 (11.92) |
| 24-hour ABPM | 133.3 (8.4) | 117.9 (8.03) |
| **Diastolic Blood Pressure in mm Hg, mean (SD)** | | |
| Research grade | 65.1 (7.6) | 74.1 (7.92) |
| Routine clinic | 71.2 (10.0) | 70.9 (9.74) |
| Awake HBPM | 74.4 (7.7) | 72.1 (6.43) |
| Awake ABPM | 75.2 (7.8) | 69.2 (6.81) |
| Asleep ABPM | 64.8 (7.9) | 61.7 (7.12) |
| 24-hour ABPM | 71.8 (6.9) | 66.7 (6.02) |
| BP: blood pressure; ABPM: ambulatory blood pressure monitoring; SD: standard deviation; IQR: interquartile range; Chronic kidney disease: estimated Glomerular Filtration rate <60 mL/min/1.73m2 | | |

**Table S3. Number and percentage of participants with mean awake BP ≥ 5 mm Hg and ≥ 10 mm Hg on HBPM vs ABPM**

|  | **Difference in mean awake blood pressure ≥ 5 mm Hg** |
| --- | --- |
| Mean SBP on HBPM – Mean SBP on ABPM, N (%) | **178 (34.9%)** |
| Mean DBP on HBPM – Mean DBP on ABPM, N (%) | **112 (21.9%)** |
|  | **Difference in mean awake blood pressure ≥ 10 mm Hg** |
| Mean SBP on HBPM – Mean SBP on ABPM, N (%) | **87 (17.1%)** |
| Mean DBP on HBPM – Mean DBP on ABPM, N (%) | **26 (5.1%)** |

SBP: systolic blood pressure, DBP: diastolic blood pressure, HBPM: home blood pressure monitoring, ABPM: ambulatory blood pressure monitoring, SD: standard deviation

**Table S4. Comparison of HBPM and ABPM in identifying uncontrolled hypertension, masked hypertension and white coat effect overall and by age, sex and history of cardiovascular disease.**

| **ABPM/HBPM** | **No on ABPM/No on HBPM** | **No on ABPM/Yes on HBPM** | **Yes on ABPM/No on HBPM** | **Yes on ABPM/Yes on HBPM** | **Overall agreement**  **(95% CI)** | **Kappa**  **(95% CI)** |
| --- | --- | --- | --- | --- | --- | --- |
| **Uncontrolled hypertension** | | | | | | |
| **Overall** | 121 (23.73%) | 64 (12.55%) | 59 (11.57%) | 266 (52.16%) | 0.75 (0.72, 0.79) | 0.47 (0.39, 0.55) |
| **Age, years** | | | | | | |
| **<73** | 65 (53.72%) | 28 (43.75%) | 24 (40.68%) | 107 (40.23%) | 0.76 (0.71, 0.82) | 0.51 (0.40, 0.63) |
| **≥73** | 56 (46.28%) | 36 (56.25%) | 35 (59.32%) | 159 (59.77%) | 0.75 (0.70, 0.80) | 0.42 (0.31, 0.54) |
| **Sex** | | | | | | |
| **Female** | 66 (54.55%) | 34 (53.55%) | 43 (72.88%) | 147 (55.26%) | 0.73 (0.68, 0.78) | 0.42 (0.31, 0.53) |
| **Male** | 55 (45.45%) | 30 (46.88%) | 16 (27.12%) | 119 (44.74%) | 0.79 (0.73, 0.84) | 0.54 (0.43, 0.65) |
| **History of cardiovascular disease** | | | | | | |
| **Yes** | 24 (19.83%) | 14 (21.88%) | 5 (8.47%) | 52 (19.55%) | 0.80 (0.71, 0.88) | 0.56 (0.39, 0.73) |
| **No** | 97 (80.17%) | 50 (78.13%) | 54 (91.53%) | 214 (80.45%) | 0.74 (0.70, 0.79) | 0.45 (0.36, 0.54) |
| **Masked uncontrolled hypertension** | | | | | | |
| **Overall** | 115 (67.25%) | 16 (9.36%) | 15 (8.77%) | 25 (14.62%) | 0.81 (0.76, 0.87) | 0.42 (0.30, 0.54) |
| **Age, years** | | | | | | |
| **<73** | 60 (52.17%) | 10 (62.50%) | 7 (46.67%) | 9 (36.00%) | 0.80 (0.71, 0.88) | 0.44 (0.26, 0.61) |
| **≥73** | 55 (47.83%) | 6 (37.50%) | 8 (53.33%) | 16 (64.00%) | 0.83 (0.75, 0.91) | 0.39 (0.24, 0.55) |
| **Sex** | | | | | | |
| **Female** | 56 (48.70%) | 12 (75.00%) | 9 (60.00%) | 14 (56.00%) | 0.76 (0.68, 0.85) | 0.34 (0.17, 0.51) |
| **Male** | 59 (51.30%) | 4 (25.00%) | 6 (40.00%) | 11 (44.00%) | 0.87 (0.80, 0.94) | 0.51 (0.35, 0.67) |
| **History of cardiovascular disease** | | | | | | |
| **Yes** | 18 (15.54%) | 3 (18.75%) | 1 (6.67%) | 16 (64.00%) | 0.87 (0.75, 0.98) | 0.46 (0.22, 0.70) |
| **No** | 97 (84.35%) | 13 (81.25%) | 14 (93.33%) | 9 (36.00%) | 0.80 (0.74, 0.87) | 0.41 (0.28, 0.54) |
| **White coat effect** | | | | | | |
| **Overall** | 290 (85.55%) | 12 (3.54%) | 20 (5.90%) | 17 (5.01%) | 0.90 (0.87, 0.93) | 0.47 (0.33, 0.61) |
| **Age, years** | | | | | | |
| **<73** | 116 (40.00%) | 4 (33.33%) | 11 (55.00%) | 7 (41.18%) | 0.89 (0.83, 0.94) | 0.49 (0.29, 0.69) |
| **≥73** | 174 (60.00%) | 8 (66.67%) | 9 (45.00%) | 10 (58.82%) | 0.91 (0.87, 0.95) | 0.44 (0.24, 0.64) |
| **Sex** | | | | | | |
| **Female** | 169 (58.28%) | 11 (91.67%) | 11 (55.00%) | 8 (52.94%) | 0.88 (0.84, 0.93) | 0.40 (0.21, 0.59) |
| **Male** | 121 (41.72%) | 1 (8.33%) | 9 (45.00%) | 9 (52.94%) | 0.92 (0.88, 0.97) | 0.58 (0.36, 0.79) |
| **History of cardiovascular disease** | | | | | | |
| **Yes** | 56 (19.31%) | 1 (8.33%) | 3 (15.00%) | 4 (23.53%) | 0.93 (0.87, 0.99) | 0.71 (0.45, 0.97) |
| **No** | 234 (80.69%) | 11 (91.67%) | 17 (85.00%) | 13 (76.47%) | 0.89 (0.86, 0.93) | 0.41 (0.25, 0.57) |

BP: blood pressure; HBPM: home blood pressure monitoring; ABPM: ambulatory blood pressure monitoring; CI: confidence interval; Uncontrolled BP defined as SBP ≥130 mm Hg or DBP ≥80 mm Hg; Masked uncontrolled BP defined as research grade SBP <130 mm Hg and research grade DBP <80 mm Hg AND mean out-of-office SBP ≥130 mm Hg or mean out-of-office DBP ≥80 mm Hg; White coat effect defined as research grade SBP ≥130 mm Hg or research grade DBP ≥80 mm Hg AND mean out-of-office SBP <130 mm Hg and mean out-of-office DBP <80 mm Hg

**Table S5. Comparison of HBPM and ABPM in identifying masked uncontrolled hypertension and white coat effect compared to routine clinic measurement.**

| **Masked uncontrolled hypertension** | | | |
| --- | --- | --- | --- |
|  | **ABPM – yes** | **ABPM – no** | **Total** |
| **HBPM – yes** | 59 | 24 | 83 |
| **HBPM – no** | 27 | 61 | 88 |
|  | 86 | 85 | 171 |
| **Overall agreement (95% CI):** | | | 0.70 (0.63, 0.77) |
| **Kappa (95% CI):** | | | 0.40 (0.26, 0.54) |
| **White coat effect** | | | |
|  | **ABPM – yes** | **ABPM – no** | **Total** |
| **HBPM – yes** | 60 | 32 | 92 |
| **HBPM – no** | 40 | 207 | 247 |
| **Total** | 100 | 239 | 339 |
| **Overall agreement (95% CI):** | | | 0.78 (0.74, 0.83) |
| **Kappa (95% CI):** | | | 0.47 (0.37, 0.58) |

BP: blood pressure; HBPM: home blood pressure monitoring; ABPM: ambulatory blood pressure monitoring; CI: confidence interval; Masked uncontrolled BP defined as research grade SBP <130 mm Hg and research grade DBP <80 mm Hg AND mean out-of-office SBP ≥130 mm Hg or mean out-of-office DBP ≥80 mm Hg; White coat effect defined as research grade SBP ≥130 mm Hg or research grade DBP ≥80 mm Hg AND mean out-of-office SBP <130 mm Hg and mean out-of-office DBP <80 mm Hg

**Table S6. Comparison of HBPM and ABPM in identifying masked uncontrolled hypertension and white coat effect overall and by age, sex and history of cardiovascular disease compared to routine clinic measurement.**

| **ABPM/HBPM** | **No on ABPM/No on HBPM** | **No on ABPM/Yes on HBPM** | **Yes on ABPM/No on HBPM** | **Yes on ABPM/Yes on HBPM** | **Overall agreement**  **(95% CI)** | **Kappa**  **(95% CI)** |
| --- | --- | --- | --- | --- | --- | --- |
| **Masked uncontrolled hypertension** | | | | | | |
| **Overall** | 61 (35.67) | 24 (14.04) | 27 (15.79) | 59 (34.50) | 0.70 (0.63, 0.77) | 0.40 (0.26, 0.54) |
| **Age, years** | | | | | | |
| **<73** | 39 (63.93) | 11 (45.83) | 10 (37.04) | 26 (44.07) | 0.75 (0.66, 0.84) | 0.50 (0.31, 0.68) |
| **≥73** | 22 (36.07) | 13 (54.17) | 17 (62.96) | 33 (55.93) | 0.64 (0.54, 0.74) | 0.28 (0.07, 0.48) |
| **Sex** | | | | | | |
| **Female** | 28 (45.90) | 13 (54.17) | 18 (66.67) | 32 (54.24) | 0.65 (0.56, 0.75) | 0.31 (0.12, 0.51) |
| **Male** | 33 (54.10) | 11 (45.83) | 9 (33.33) | 27 (45.76) | 0.75 (0.65, 0.84) | 0.49 (0.30, 0.68) |
| **History of cardiovascular disease** | | | | | | |
| **Yes** | 8 (13.11) | 6 (25.00) | 2 (6.45) | 15 (25.42) | 0.74 (0.58, 0.89) | 0.46 (0.15, 0.77) |
| **No** | 53 (86.89) | 18 (75.00) | 25 (92.59) | 44 (74.58) | 0.69 (0.61, 0.76) | 0.38 (0.02, 0.53) |
| **White coat effect** | | | | | | |
| **Overall** | 207 (61.06) | 32 (9.44) | 40 (11.80) | 60 (17.70) | 0.78 (0.74, 0.83) | 0.47 (0.37, 0.58) |
| **Age, years** | | | | | | |
| **<73** | 81 (39.13) | 14 (43.75) | 17 (42.50) | 26 (43.33) | 0.77 (0.70, 0.84) | 0.46 (0.30, 0.62) |
| **≥73** | 126 (60.87) | 18 (56.25) | 23 (57.50) | 34 (56.67) | 0.79 (0.74, 0.85) | 0.48 (0.34, 0.61) |
| **Sex** | | | | | | |
| **Female** | 115 (55.56) | 25 (78.13) | 21 (52.50) | 38 (63.33) | 0.76 (0.71, 0.82) | 0.45 (0.32, 0.59) |
| **Male** | 92 (44.44) | 7 (21.88) | 19 (47.50) | 22 (36.67) | 0.81 (0.74, 0.87) | 0.50 (0.34, 0.67) |
| **History of cardiovascular disease** | | | | | | |
| **Yes** | 37 (17.87) | 3 (9.38) | 8 (20.00) | 16 (26.67) | 0.82 (0.73, 0.92) | 0.61 (0.41, 0.81) |
| **No** | 170 (82.13) | 29 (90.63) | 32 (80.00) | 44 (73.33) | 0.77 (0.72, 0.82) | 0.43 (0.31, 0.55) |

Masked uncontrolled BP defined as research grade SBP <130 mm Hg and research grade DBP <80 mm Hg AND mean out-of-office SBP ≥130 mm Hg or mean out-of-office DBP ≥80 mm Hg; White coat effect defined as research grade SBP ≥130 mm Hg or research grade DBP ≥80 mm Hg AND mean out-of-office SBP <130 mm Hg and mean out-of-office DBP <80 mm Hg

**Table S7. Agreement of HBPM and ABPM in identifying uncontrolled blood pressure, masked uncontrolled hypertension and white coat effect.**

| **Uncontrolled hypertension** | | | |
| --- | --- | --- | --- |
|  | **ABPM – yes** | **ABPM – no** | **Total** |
| **HBPM – yes** | 5 | 2 | 7 |
| **HBPM – no** | 0 | 503 | 503 |
| **Total** | 5 | 505 | 510 |
| **Overall agreement (95% CI):** | | | 0.99 (0.99, 1.00) |
| **Kappa (95% CI):** | | | 0.83 (0.60, 1.00) |
| **Masked uncontrolled hypertension** | | | |
|  | **ABPM – yes** | **ABPM – no** | **Total** |
| **HBPM – yes** | 42 | 30 | 72 |
| **HBPM – no** | 34 | 144 | 178 |
|  | 76 | 174 | 250 |
| **Overall agreement (95% CI):** | | | 0.74 (0.68, 0.79) |
| **Kappa (95% CI):** | | | 0.38 (0.26, 0.51) |
| **White coat effect** | | | |
|  | **ABPM – yes** | **ABPM – no** | **Total** |
| **HBPM – yes** | 18 | 16 | 34 |
| **HBPM – no** | 15 | 211 | 226 |
| **Total** | 33 | 227 | 260 |
| **Overall agreement (95% CI):** | | | 0.88 (0.84, 0.92) |
| **Kappa (95% CI):** | | | 0.46 (0.30, 0.62) |

BP: blood pressure; HBPM: home blood pressure monitoring; ABPM: ambulatory blood pressure monitoring; CI: confidence interval; Uncontrolled BP defined as SBP ≥140 mm Hg or DBP ≥90 mm Hg for research grade SBP and mean out-of-office SBP ≥135 mm Hg or mean out-of-office DBP ≥85 mm Hg; Masked uncontrolled BP defined as research grade SBP <140 mm Hg and research grade DBP <90 mm Hg AND mean out-of-office SBP ≥135 mm Hg or mean out-of-office DBP ≥85 mm Hg; White coat effect defined as research grade SBP ≥140 mm Hg or research grade DBP ≥90 mm Hg AND mean out-of-office SBP <135 mm Hg and mean out-of-office DBP <85 mm Hg

**Figure S1A. Histogram of the difference of mean awake SBP on HBPM versus mean awake SBP on ABPM.**

SBP: systolic blood pressure; HBPM: home blood pressure monitoring; ABPM: ambulatory blood pressure monitoring
The red line portrays a normal distribution with a mean of 0.84 and standard deviation of 10.97.
x-axis presents the mean difference of SBP: HBPM SBP – ABPM SBP

**Figure S1B. Histogram of the difference of mean awake DBP on HBPM versus mean awake DBP on ABPM.**

DBP: diastolic blood pressure; HBPM: home blood pressure monitoring; ABPM: ambulatory blood pressure monitoring
The red line portrays a normal distribution with a mean of 0.07 and standard deviation of 6.41.
x-axis presents the mean difference of DBP: HBPM DBP – ABPM DBP
